# Fetus of 8q22.2q24.3 duplication and 13q33.2q34 deletion derived from a maternal balanced translocation

**DOI:** 10.1111/jog.14386

**Published:** 2020-07-08

**Authors:** Tong Liu, Huihui Xie, Jingbo Zhang, Xia Wang, Jing Sha, Jingfang Zhai

**Affiliations:** ^1^ Department of Prenatal Diagnosis Medical Center, XuZhou Central Hospital XuZhou Clinical School of Xuzhou Medical University Jiangsu China

**Keywords:** 13q deletion, 8q duplication, copy number variations sequencing, noninvasive prenatal testing, ultrasonography

## Abstract

The concomitant occurrence of 8q duplication and 13q deletion is the first to be detected by noninvasive prenatal testing (NIPT) to date. Through case analysis, we could provide a clinical approach to pregnant women with chromosomal abnormalities revealed by NIPT. The combination of traditional karyotype and copy number variation sequencing (CNV‐seq) could better locate the abnormal chromosomal region and further identify the source of fetal chromosomal abnormalities. Simultaneously, we evaluated the fetal morphology by ultrasound examination. The karyotype of the fetus was 46,XY,der(13)t(8;13)(q22;q32)mat and CNV‐seq results showed that there was an approximately 45.26‐Mb duplication in 8q22.2‐q24.3 (101040001–146 300 000) and an approximately 9.54‐Mb deletion in 13q33.2‐q34 (105560001–115 100 000). Prenatal ultrasound revealed the fetal structural abnormalities presented with hypoplasia of the cerebellar vermis, a flat nose, echogenic bowel and absent gallbladder. Herein, we consider that combination detection of traditional karyotyping, CNV‐seq and ultrasonography provides a valuable method for pregnant women with abnormal NIPT.

## Introduction

Recently, noninvasive prenatal testing (NIPT) has been increasingly applied to clinical practice and deserved further promotion due to the reduced price in China. However, the clinical application of NIPT in subchromosomal abnormalities is limited because the results of NIPT are affected by the mother, fetus and placenta, with false positive and inaccuracy. In a retrospective study of 117 pregnant women, the sensitivity and specificity of the conventional NIPT method for detecting copy number variations (CNV) larger than 1 Mb were 61.1% and 95.0%, respectively, with a false‐positive rate of 5.0%.^1^ In another study, the sensitivity and specificity of CNVs over 6 Mb revealed by NIPT were 83% and 99.6%, respectively, while the positive‐predictive value was 55%.^2^ Therefore, NIPT is a screening test and further diagnostic means is needed. Positive NIPT results indicate an increasing trend of abnormal karyotypes including different segments of duplication and deletion, which could discover the presence of a heterotopic chromosome in either of the parents.^3^, ^4^, ^5^ Whether chromosomal abnormalities are inherited or mutated, fetuses with unbalanced genetic material may have adverse clinical outcomes. Therefore, genetic confirmation is necessary for such fetuses. The resolution of the traditional karyotype is low, but it can identify the balanced translocation and other specific chromosome structures at a low cost. In contrast, CNV sequencing (CNV‐seq) by next‐generation sequencing technology not only has a higher resolution and effectively detects microdeletion and microduplication of chromosome, but also possesses the advantages of high specificity and sensitivity，which can provide diagnostic support for the fetuses of structural abnormalities in prenatal ultrasound and positive NIPT results. For CNV larger than 100 kb, the specificity and sensitivity of CNV‐seq were > 99% and compared with chromosomal microarray analysis, the concordance rate of the two test results was 100%.^6^ However, CNV‐seq fails to detect structural chromosomal abnormalities and low levels of chromosomal mosaicism. Therefore, the combined application of karyotype analysis and CNV‐seq can significantly analyze the nature and origin of chromosome abnormalities in prenatal diagnosis. Here, we report one case with abnormal NIPT by conventional chromosome analysis techniques and CNV‐seq. At the same time, according to the CNV‐seq results of molecular genetics, multiple abnormalities of ultrasound examination were analyzed.

## Case Presentation

**Figure 1:**
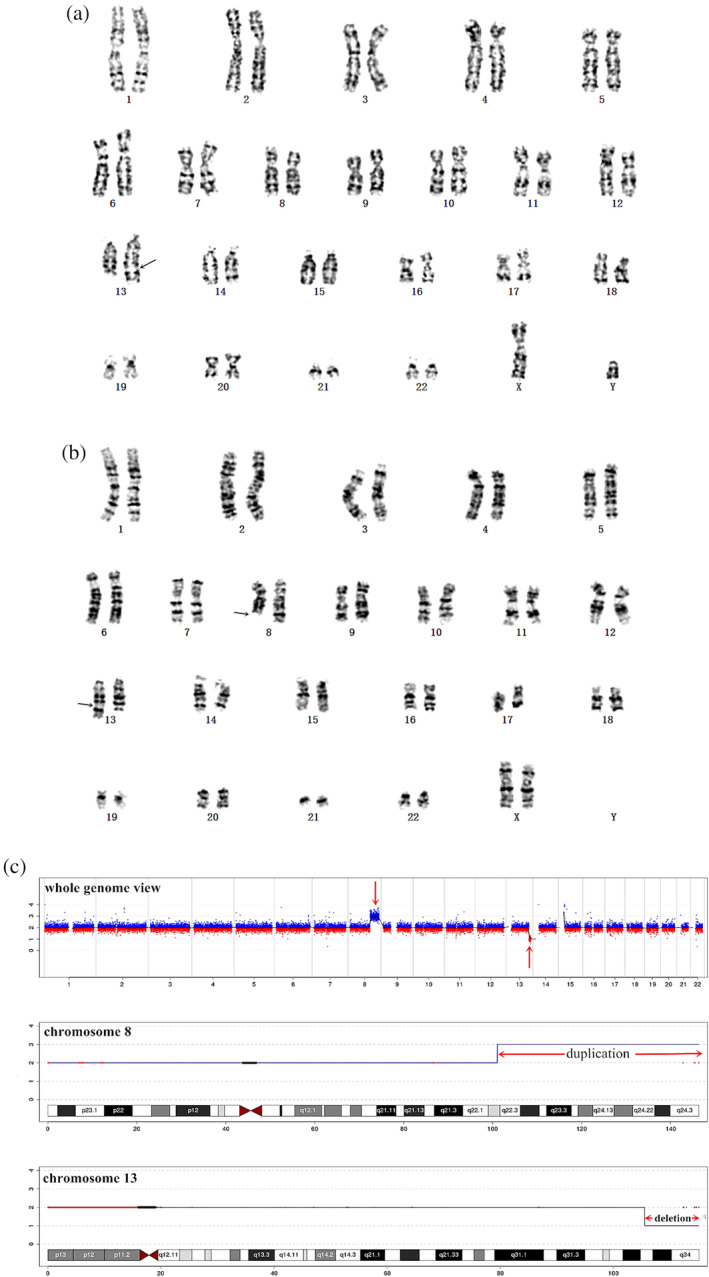
(a) The fetal karyotype was 46,XY,add(13)(q31) at the level of 300–400 bands. (b) The fetal results of copy number variation sequencing (CNV‐seq) showed a 45.26‐Mb duplication in 8q22.2‐q24.3 (101040001–146300000) and a 9.54‐Mb deletion in 13q33.2‐q34 (105560001–115100000). (c) The peripheral blood karyotype of pregnancy women was 46,XX,t(8;13)(q22;q32) at the level of 300–400 bands.

**Figure 2:**
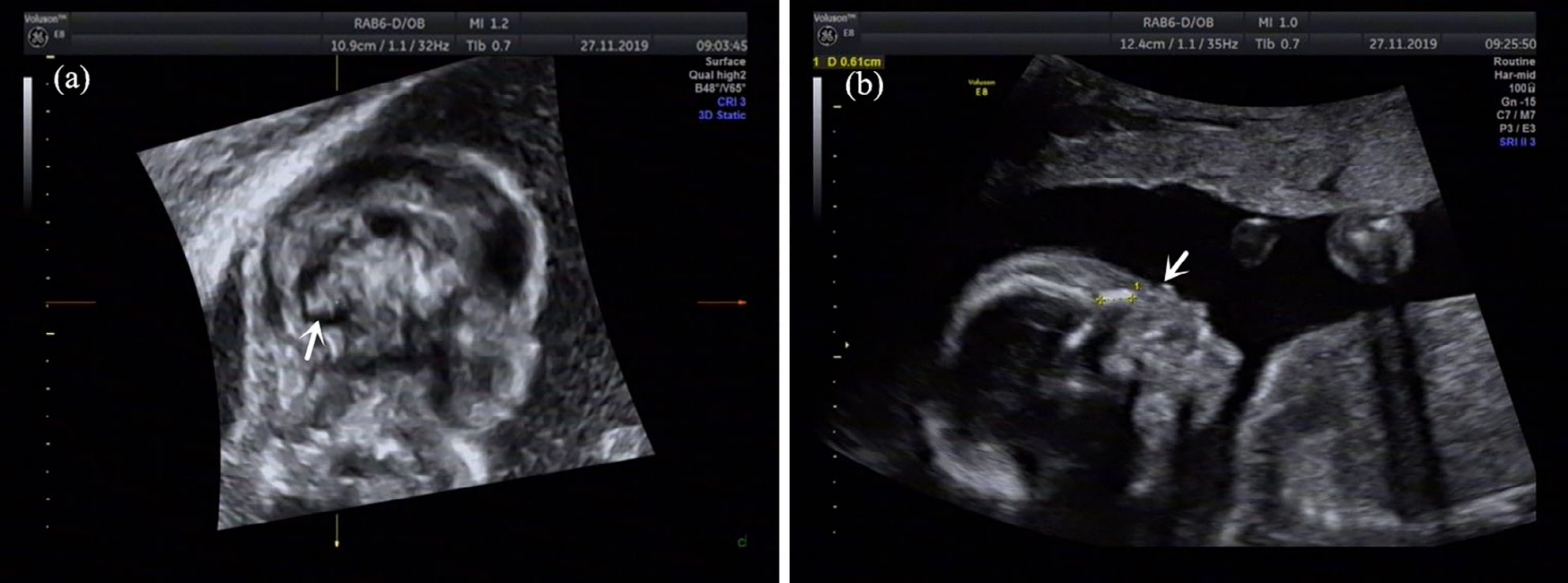
The fetal ultrasound imaging showed hypoplasia of the cerebellar vermis (a) and a flat nose (b).

A 30‐year‐old healthy primigravida woman was referred to prenatal diagnosis medical center of Xuzhou Central Hospital. She had no history of adverse reproduction and drug usage with non‐consanguineous marriage. The result of NIPT indicated partial trisomy 8 and deletion in chromosome 13 at 18‐week gestation. The woman chose the amniocentesis for karyotype analysis and CNV‐seq in the following day. The fetal karyotype was 46,XY,add(13)(q31) (Fig. 1a). In addition, the result of CNV‐seq showed that there was an approximately 45.26‐Mb duplication in 8q22.2‐q24.3 (101040001–146300000) and an approximately 9.54‐Mb deletion in 13q33.2‐q34 (105560001–115100000) (Fig. 1b). Prenatal ultrasound at 21‐week gestation showed abnormal fetal structures presented with hypoplasia of the cerebellar vermis (Fig. 2a), a flat nose (Fig. 2b), echogenic bowel and absent gallbladder. Moreover, patient's peripheral blood lymphocytes were suggested to detect, mainly to further clarify the origin of the derived chromosome. The mother's karyotype revealed a balanced translocation between chromosomes 8 and 13, carrying the following karyotype 46,XX,t(8;13)(q22;q32) (Fig. 1c), while the father's was normal. Finally, the couple opted to terminate the pregnancy after being fully informed of the adverse pregnancy outcomes. Amniotic fluid of the fetus and peripheral blood of the parents were collected for karyotype analysis and CNV‐seq after informed consent. This study was approved by Xuzhou Central Hospital Ethics Committee.

## Discussion

Derivative chromosomes are often caused by unbalanced chromosome rearrangements that appear spontaneously or may be inherited from healthy parents carrying balanced reciprocal translocations. In this paper, we report the fetus with 8q duplication and 13q deletion resulting from a maternal balanced translocation. Therefore, the karyotype of the fetus is reassigned as 46,XY,der(13)t(8;13)(q22;q32)mat which appears to be responsible for the multiple abnormalities according to the reported literatures and a large amount of data. On the other hand, the result of CNV‐seq shows a terminal q22.2‐q24.3 (101040001–146300000) duplication of approximately 45.26‐Mb. Isolated duplication of 8q22.2‐q24.3 is responsible for dysmorphic features such as hypertelorism, microretrognathia, telecanthus, upturned and broad nose, cleft palate and a variable degree of intellectual disability,^7^ however, the duplication of segment is not associated with fetal changes in our ultrasound imaging. Relatedly, a female fetus with absent gallbladder and other multiple congenital anomalies was found to have partial trisomy 8 chromosome on karyotype analysis via amniocentesis.^8^ The region of q22.2‐q24.3 encompasses 47 morbid genes (https://decipher.sanger.ac.uk/browser) and no gene variants related to absent gallbladder have been found. Among these, *TAF2*(OMIM: 604912), *KCNK9*(OMIM: 605847), *TRAPPC9*(OMIM: 611966), and *RECQL4*(OMIM: 603780) have been linked to the intellectual disability, while other genes such as *SAMD12*(OMIM: 618073), *KCNQ3*(OMIM: 602232) and *GPAA1*(OMIM: 603048)have been associated with epilepsy.^9^, ^10^

Hence, we speculate that the various malformations of the fetus in ultrasound may be mainly the result of distal deletion in 13q, among which, the cerebellar vermis hypoplasia is a severe abnormality. The 13q terminal deletion is mainly characterized by intellectual disability, developmental delay and congenital anomalies of the brain, such as Dandy–Walker malformation (DWM), corpus callosum agenesis, encephalocele and holoprosencephaly; facial deformity and other disorders, such as bowel atresia and lung hypoplasia.^11^, ^12^ Within the 13q terminal area, 13q32.2q33.1 is a critical region for cerebellar dysgenesis that has been confirmed to be related to the haploinsufficiency of *ZIC2* and *ZIC5*, located in 13q32.2‐q32.3.^11^, ^13^ The CNV that encompass many functional genes may give rise to the congenital malformation by regulating genes haploinsufficiency, changing gene regulation and other mechanism. Differently, distal 13q deletion can occur due to disruption of multiple genes, other than haploinsufficiency of *ZIC2* and *ZIC5*.^14^ Bellucco *et al*. described two patients with right cerebellar hypoplasia, carrying an 11.9‐Mb deletion in the 13q33.1‐q34 region and a 3.8‐Mb in 13q34, respectively.^15^ Chen *et al*. also reported a fetus who carried a 13q33.3‐ter deletion had DWM based on 24‐week ultrasound.^16^ Herein, We identify a 9.54 Mb (105560001–115100000) deletion of 13q33.2‐q34 region, which is consistent with these literatures. Therefore, we speculate that there are other important genes associated with cerebellar vermiform hypoplasia in the 13q33.2‐q34 region. Or the function of known genes related to cerebellar vermis dysplasia has not been found. To our knowledge, the 13q33.2‐q34 region contains many important genes (https://decipher.sanger.ac.uk/browser), which has no clear evidence connected with the cerebellar vermis hypoplasia in human currently. Of these genes, the genes with relevance to disease include *EFNB2*(OMIM: 600527), *ARHGEF7*(OMIM: 605477), *CHAMP1*(OMIM: 616327), *COL4A1*(OMIM: 120130) and *COL4A2*(OMIM: 120090). The gene *EFNB2* located in 13q33.2 plays an important role in genital development and congenital eye malformations. As a candidate gene for genital malformations, loss of function variant in the *EFNB2* gene exhibits hypospadias and ambiguous genitalia.^17^ The gene *ARHGEF7* mapped in 13q34 is suggested as a candidate gene for psychomotor developmental delay, whose haploinsufficiency can contribute to the epileptic phenotype.^18^ In addition, *CHAMP1* encodes a protein with a function in kinetochore‐microtubule attachment and in the regulation of chromosome segregation, both of which are known to be important for neurodevelopment. Variants in *CHAMP1* will give rise to intellectual disability with severe speech impairment, motor developmental delay, muscular hypotonia and similar dysmorphic features including short philtrum and a tented upper and everted lover lip, which is related with the loss of important zinc‐finger domains.^19^ *COL4A1* and *COL4A2* are present in almost all basement membranes. Allelic heterogeneity caused by *COL4A1* and *COL4A2* variants results in brain abnormalities manifesting with schizencephaly and severe neurological deficits including cerebral palsy, intellectual disability and focal epilepsy,^20^ which has only been demonstrated in a series of mutant mouse lines. The parents finally terminated the pregnancy and refused to conduct an autopsy, so we could not appreciate the detailed characterizations and mental status.

The Published literatures found that there were two similar cases with accompanying the appearance of 8q duplication and 13q deletion derived from the maternal balanced translocation (Table 1). The first case showed partial trisomy 8q from a maternal carrier of a t(8;13)(q21;q34), and the phenotype was dominated by partial trisomy 8q.^21^ This case did not describe the microdeletion of chromosome 13, lacking the molecular mapping of breakpoints and gene content that were limited to genomic techniques. The second case had de novo partial trisomy 8q (56.8 Mb from 8qter) and partial monosomy 13q (0.28 Mb from 13qter) from a der(13)t(8;13)(q21.3;q34). The congenital malformations might be mainly the result of trisomy 8q.^22^ In this case, the genes in the duplication region of 8q21.3q24.3 were not mentioned. *GRTP1*(RefSeq: NM_024719), *ADPRHL1*(OMIM: 610620), DCUN1D2(RefSeq. ID: NM_018185), TMCO3(OMIM: 617134) and *TFDP1*(OMIM: 189902) genes in the deletion region of 13q34 were introduced briefly, disappointingly, which had no significant relationship with the described congenital malformations. In contrast, in our case, the deletion region of 13q was much larger. Despite there were similar duplication and deletion in our case, the clinical phenotypes were completely different and we thought a deletion might have more important influence than a duplication according to the theory of heredity, depending on the function of the genes in that region.

**Table 1:** Review of case reports associated with the 8q duplication and 13q deletion

	Diagnosis	NIPT	Karyotype	Origin	Gene content and coordinate	Outcome	Clinical findings
First case Abuelo, D	Postnatal	ND	46,XY,der (13),t(8;13) (q21;q34)	Maternal balanced translocation	NS	Born and follow up to 11 months	Ocular hypertelorism, exotropia, a double skin crease below the lower eyelid, small pinnae with posterior angulation, pugged nose and long philtrum, micrognathia, short neck, marrow pelvis, hypoplastic scrotum and occurring a generalized tonic seizure
Second case Sohn Y B	Postnatal	ND	46,XX, der(13)t(8;13)(q21.3;q34)	Maternal balanced translocation	del(13)(q34) 0.28 Mb (arr 113986985‐114 266 156) dup (8)(q21.3q24.3) 56.8 Mb (arr 88908177‐145 788 550)	Born and follow up to 37 days	Corpus callosum agenesis, crumpled ear, hypertelorism, micrognathia, cleft palate, and overlapping fingers/toes and congenital heart disease
Present case	Prenatal	Partial trisomy 8 and deletion in 13 chromosome	46,XY,der(13)t(8;13)(q22;q32)	Maternal balanced translocation	del (13)(q33.2q34) 9.54 Mb (arr 105560001–1151000000) dup (8)(q22.2q24.3) 45.26 Mb (arr 101040001–146300000)	Termination of pregnancy at 21 weeks	Ultrasound examination showed abnormal fetus presented with microcephalic, a depressed nasal bridge, echogenic bowel and absent gallbladder

ND, not done; NS, not stated.

In a sense, the woman was fortunate to find herself as a carrier of balanced translocation in the first pregnancy without experiencing adverse pregnancy outcomes such as spontaneous miscarriage, stillbirth, or congenital malformations in live‐born progeny, which had guiding significance for future pregnancy. In genetic counseling, we suggest that the couple may choose natural pregnancy, which might cause likely adverse pregnancy outcomes again. On the other hand, she may also opt for preimplantation genetic diagnosis to improve the likelihood of achieving a successful pregnancy.

In conclusion, we use traditional karyotype and CNV‐seq to analyze the patients with positive NIPT results, consisting of the exchange of the terminal chromosome segments. At the same time, ultrasonography can evaluate the malformations of the fetus timely. We believe the combination detection of traditional karyotyping, CNV‐seq and ultrasonography can become a key step in such cases, complementing valuable details that are useful for a more precise phenotype characterization of each individual and for estimating their prognosis, but also for expanding our knowledge regarding this rare disease.

## Disclosure

None declared.
